# HIV cure research contributions from Africa in the last three decades

**DOI:** 10.3389/fimmu.2025.1576667

**Published:** 2025-08-08

**Authors:** Damalie Nakanjako, Edward N. Kankaka, Cynthia Lungu, Ronald M. Galiwango, Steven J. Reynolds, Tokameh Mahmoudi, Thumbi Ndung’u

**Affiliations:** ^1^ Department of Medicine, School of Medicine, Makerere University College of Health Sciences, Kampala, Uganda; ^2^ Infectious Diseases Institute, Makerere University College of Health Sciences, Kampala, Uganda; ^3^ Africa-Europe Cluster of Excellence in Translational Science in Infection, Immunity and Inflammation (Co-Trii), Makerere University College of Health Sciences, Kampala, Uganda; ^4^ Rakai Health Sciences Program, Masaka, Uganda; ^5^ Department of Pathology, Erasmus Medical Centre, Rotterdam, Netherlands; ^6^ Division of Intramural Research, National Institute of Allergy and Infectious Diseases, National Institutes of Health, Bethesda, MD, United States; ^7^ Department of Urology, Erasmus Medical Centre, Rotterdam, Netherlands; ^8^ Africa Health Research Institute, Durban, South Africa; ^9^ HIV Pathogenesis Programme, The Doris Duke Medical Research Institute, University of KwaZulu-Natal, Durban, South Africa; ^10^ Ragon Institute of Massachusetts General Hospital, Massachusetts Institute of Technology and Harvard University, Cambridge, MA, United States; ^11^ Division of Infection and Immunity, University College London, London, United Kingdom

**Keywords:** HIV cure, biomedical research, research and discovery, Africa, non-B HIV subtypes

## Abstract

**Introduction:**

The HIV epidemic in Africa is characterized by extensive viral subtype diversity and human genetic heterogeneity which influence disease outcomes; amidst the co-morbidities that modulate HIV reservoirs and immune responses. This paper provides an overview of the quantity and spectrum of HIV cure research in context of the contributions made by African scientists toward HIV cure related research in Africa.

**Methods:**

Using a hybrid environmental scan, we searched the Treatment Action Group website to identify registered HIV cure-related observational and interventional studies between 1995-2024. To identify published papers related to HIV or SIV latency, we searched PubMed for articles with HIV or SIV in the title PLUS terms related to virus latency in the title or in medical subject headings (MeSH); and downloaded results in PubMed format in a text file. We used an R script which checked NCBI to identify articles which cited the original paper that first described the HIV reservoir in 1995, restricting to only those within the query result. This was repeated using loop functions until we obtained all articles directly or indirectly linked to the original paper.

**Results:**

Overall, we show an increasing trend of HIV cure-related observational and interventional studies globally; with the least number of studies in Africa. The PubMed query retrieved 7122 HIV cure-related published articles, as at 23 July 2024; of which 2820 were directly or indirectly linked to understanding the HIV reservoir. Of the 2916 articles with first author affiliation country determined, only 52 (0.02%) had affiliations from African institutions. Of the 1955 articles with last author affiliation country determined, only 43 (0.02%) had affiliations from institutions in Africa. The majority of articles with first or last authors from African institutes were descriptive clinical studies of HIV infection, with less than ten studies specifically addressing HIV latency.

**Conclusion:**

Scale up of HIV cure research in Africa remains critical to hasten achievement of the global goal of an end to the AIDS epidemic by 2030. There is a need to bridge the technical, infrastructural and technological divides and address constraints in funding and capacity; to promote discovery, characterization and application of promising innovative therapies including immunotherapies and cell and gene therapies towards attaining an effective, durable, affordable and scalable HIV cure.

## Introduction

Africa has the highest burden of HIV infection, with more than 70% of people living with HIV (PLHIV) (1). The HIV epidemic in Africa is characterized by extensive viral subtype diversity and human genetic heterogeneity which influence immunological and disease outcomes; amidst the unique co-morbidities that modulate HIV reservoirs and immune responses (2–5). Most reservoir studies and cure interventions have however focused on clade B in high income countries. The human immunodeficiency virus type 1 (HIV-1), a member of the lentivirus subfamily originating from the simian immunodeficiency virus (SIV), infects both dividing and non-dividing cells, and following reverse transcription of the viral RNA genome, integrates into the host chromatin where it can persist for the cell’s lifetime if the cell enters a latent state. Persistence of the latent reservoir within multiple immune cells during suppressive antiretroviral therapy (ART) is the major obstacle to curing HIV (6, 7). Most factors governing viral latency remain unresolved, particularly in non-B HIV subtypes that predominate the epidemic in Africa in the context of current antiviral treatment regimens that are ineffective at eliminating reservoirs of latent HIV (8). Moreover, the HIV epidemic in Africa is different in several ways including the prevalent non-B HIV subtypes, vertical mother-to-child transmission, heterosexual transmission, as well as female predominance with over two-thirds of the infections among women of reproductive and post-menopausal age-groups (9); all of which may influence HIV persistence and latent HIV reservoir sizes (10, 11).

Early ART followed by combination immunotherapies has shown promise for durable HIV control following ART interruption, but remission is variable and the underlying immune mechanisms and predictors of reservoir control remain poorly understood (12–15). Moreover, HIV-associated inflammation and immune dysfunction persist despite suppressive ART in chronic HIV treatment cohorts in SSA (16–21). Compounding these gaps in knowledge, is the limited contribution of HIV cure research in Africa; a continent which hosts two-thirds of the epidemic with pre-dominant non-B HIV subtypes that are not yet well understood. HIV cure related studies in Africa are mostly descriptive clinical studies (22–25) and a few drug treatment clinical trials focused on clinical presentation of HIV and co-infections including tuberculosis, hepatitis and several opportunistic infections among individuals with severe HIV disease.

Similarly, ‘shock and kill’ strategies, which aim to eliminate HIV latently infected CD4+ T cells using latency reversal agents (LRAs) that reactivate HIV expression rendering infected cells susceptible to elimination by cytopathic effects or clearance by HIV-specific cytotoxic T cells, have also been extensively investigated, but mostly in context of HIV subtype B. Such interventions inform the exploration of alternative strategies that achieve durable suppression of viremia in the absence of ART, and or elimination of HIV latently infected cells (26); that would need to first be investigated and scaled to non-B subtypes in Africa. In addition, the “block-and-lock” strategies aiming to permanently silence all proviruses even after treatment interruption (27) need to be contextualized within known characteristics of the latent HIV reservoir among non-B subtype infections that are predominant in African men and in women. Therefore, a better understanding of HIV-1 latency and reservoir dynamics among HIV-1 non-B subtypes in Africa is required to facilitate the development of rational strategies for HIV remission or eradication, and to inform development of therapeutic vaccines and adjunctive immunotherapies; as a strategy towards an effective, durable, affordable and scalable HIV cure for all PLHIV (28).

This paper provides an overview of the quantity and spectrum of HIV cure research in Africa and describes the contribution of African scientists to HIV cure related research between 1995, when the HIV reservoir was first described (29), and 2024. We also identify opportunities to accelerate HIV cure research in sub-Saharan Africa. Our findings inform the development of strategic approaches to reduce the inequities that slow down involvement in HIV biomedical HIV cure research; and aim to bolster involvement of Africa in the global research priorities for an HIV cure (28).

## Methods

We conducted a hybrid environmental scan that involved multiple information sources relevant to HIV cure research in Africa, beyond published academic literature.

To identify the registered HIV cure-related observational and interventional studies between 1995-2024, we used the Treatment Action Group website which maintains a list of registered completed or ongoing observational studies or clinical trials related to HIV cure research (30). Registered studies are listed whether results are published or not. We extracted this list into an excel spreadsheet and obtained study locations as well as start and end dates of these studies; or used the registry numbers to obtain this information from clinicaltrials.org (where applicable). The start and end dates were used to count active observational and interventional studies per year in each continent. We also used bar plots to visualize who funds these studies in Africa alone and globally.

To identify published papers related to HIV or SIV latency between 1995-2024, we searched PubMed for articles with HIV or SIV in the title PLUS terms related to virus latency in the title or in medical subject headings (MeSH); and downloaded results in PubMed format in a text file. The search query used was: ((HIV[Title] OR Human Immunodeficiency Virus[Title] OR SIV[Title] OR Simian Immunodeficiency Virus[Title]) AND ((Virus Integration[MeSH Terms] OR Virus Activation[MeSH Terms] OR Virus Latency[MeSH Terms] OR Proviruses[MeSH Terms]) OR (Virus Integration[Title] OR Virus Activation[Title] OR Virus Latency[Title] OR latent[Title] OR latency[Title] OR reservoir[Title] OR provirus[Title] OR proviral[Title] OR proviruses[Title] OR persistent[Title] OR persistence[Title]))). Among the articles returned (query result), we selected the original paper which first described the HIV reservoir (29). Subsequently, we used an R script which checked NCBI to identify articles which cited the original paper, restricting to only those within the query result. This was repeated using loop functions to obtain other articles which cited the selected articles, until we obtained all articles directly or indirectly linked to the original paper. These were used in downstream analyses as described below.

### Contribution of African scientists

We created an R script which extracted PMID, publication year, authors, author affiliations (if available); and country from the author affiliation. Next, we matched each country to countries in the world map from the *rnaturalearch* package in R and obtained the respective continent. Imperfect matches were manually corrected to retrieve country and continent, ensuring all Africa-related papers were correctly identified. Bar plots were used to represent counts of authors in each year (1995 to 2024), and this was repeated to show trends in first author contributions, last author contributions, and contribution from any author; in all continents and for Africa only.

### Intra and inter-continental collaborations

To assess publishing networks (who is publishing with who), we selected only articles with more than two authors and where author affiliation countries were available. We then used a matrix to count pairwise collaborations between authors in different continents. For example, if a paper included authors from Africa, Oceania, and North America, this contributed an Africa-Oceania pair, Africa-North America pair, and an Oceania-North America pair. Where a paper lists affiliations on only one continent, that contributes one pair within that continent (e.g., Africa-Africa). The result was visualized in a chord diagram where the thickness of a chord connecting two continents represents the magnitude of that collaboration.

### Impact of work involving African-affiliated researchers

We used the citations information (papers citing other papers) to estimate the number of citations per year (denominator was years since publication) as a proxy for impact; and used a ridgeline plot from the *ggridges* package in R to compare impact of work by first or last authors with affiliations in Africa with the impact of work by first or last authors with affiliations in other continents.

In the sub-analyses described below, we selected only articles with first or last author affiliation in an African country.

### Spectrum of HIV cure-related research in Africa (what the work involving African-affiliated researchers was about)

We extracted medical subject headings where available in the PubMed-format query result and filtered for major MeSH qualifiers. For example, for the Mesh term “MH - *Proviruses/genetics/immunology”, we based on the asterisk (*) to select *Proviruses* as a major topic addressed in the article. The frequency of these major topics was represented in bar plots. We also extracted publication types (e.g., “PT - Review”) and similarly represented these in bar plots.

### Funding for HIV-related research in Africa (who funded the published work involving African-affiliated researchers)

We extracted grants information (e.g., “GR - P01 AI099783-01/AI/NIAID NIH HHS/United States”), filtered for institution and country (e.g., “NIAID NIH HHS/United States”) and represented the frequency of funding for listed agencies in bar plots.

## Results

### Registered observational and interventional studies

**Figure 1:**
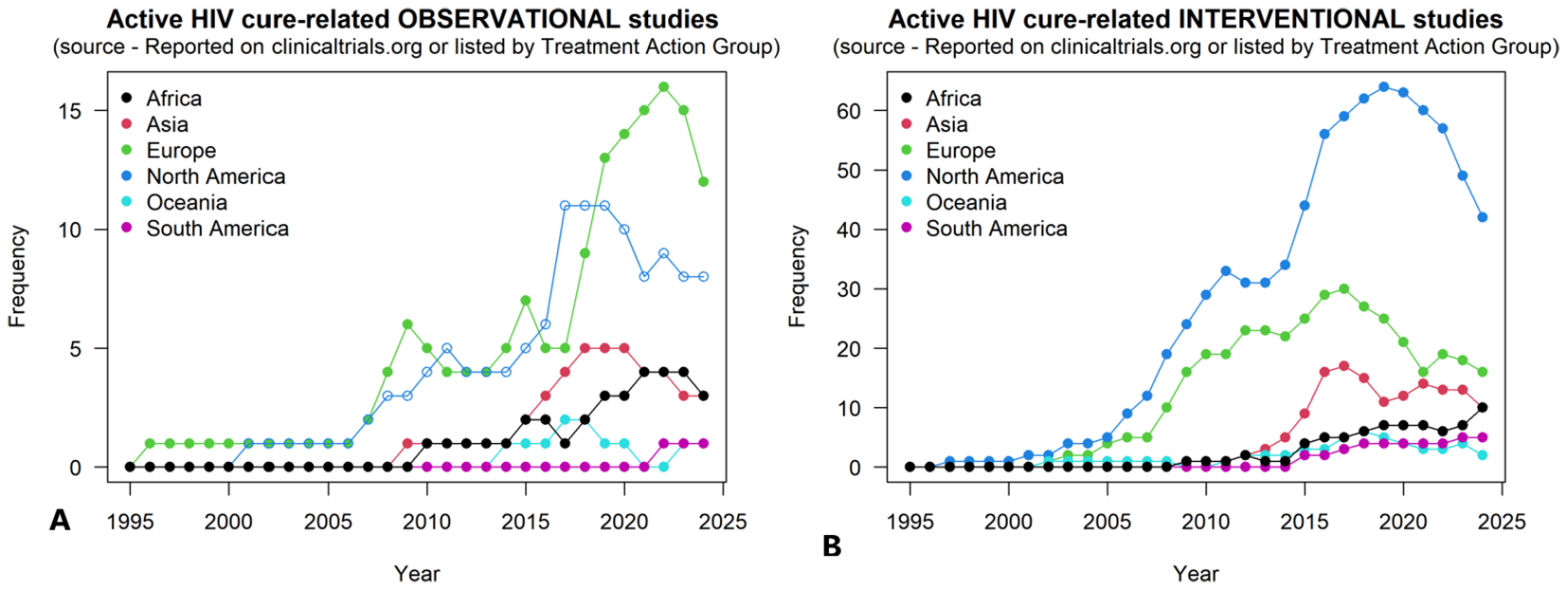
Plot of registered HIV cure-related observational and interventional studies between 1995-2024: **(A)** shows HIV cure-related observational studies and **(B)** shows HIV cure-related interventional studies.

Overall, we show an increasing trend of HIV cure related observational and interventional studies globally; with the least number of studies in Africa (**Figure 1**). The PubMed query retrieved 7122 HIV cure related published articles, as of 23 July 2024, of which 2820 were linked to understanding the HIV reservoir.

### Contribution of African scientists

**Figure 2:**
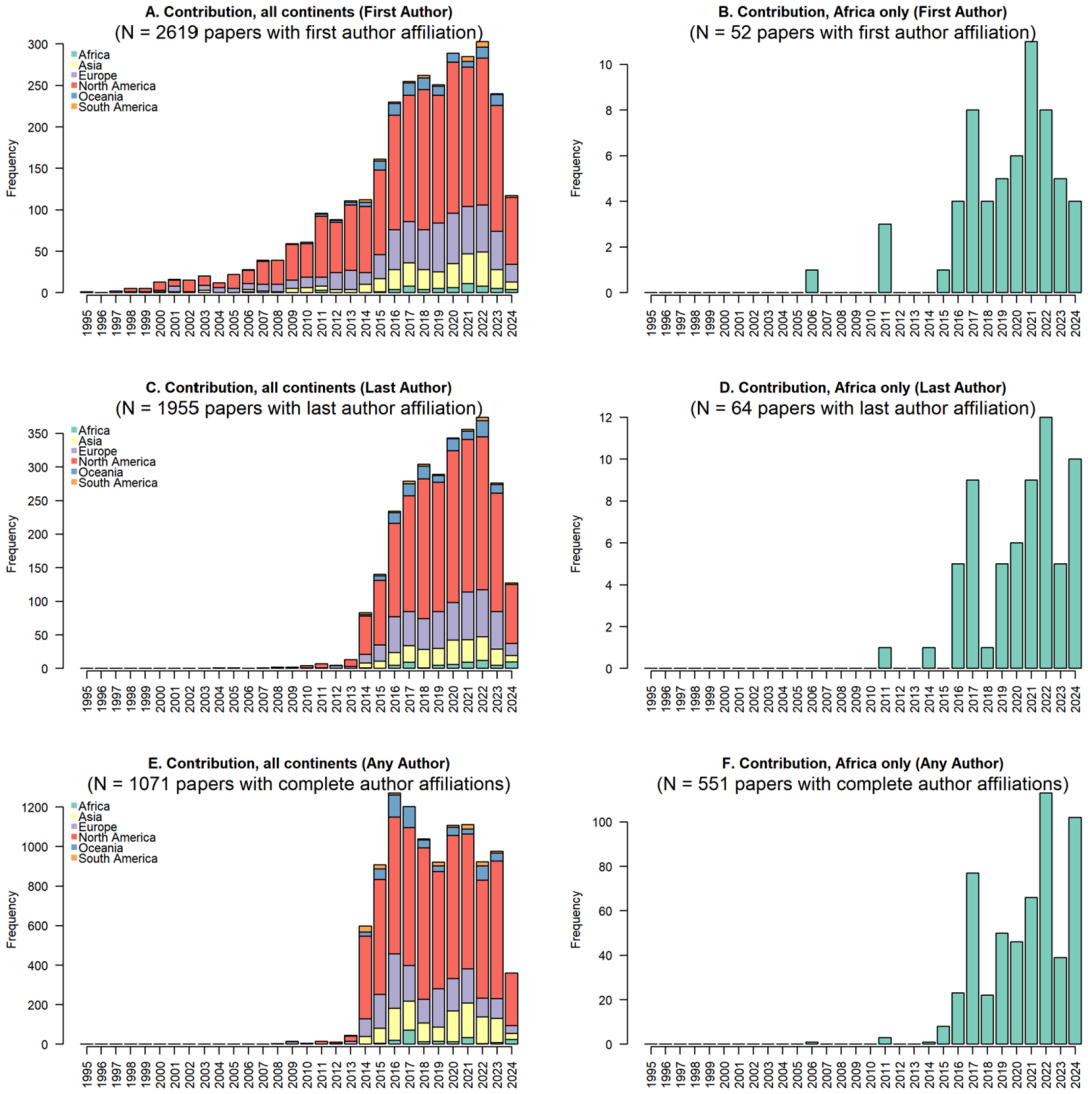
Contribution of scientists in Africa towards HIV cure related publications between 1995-2024. **(A)** shows contributions from all continents, **(B)** shows fist-author contributions from Africa, **(C)** shows last author contributions from all continents, **(D)** shows last author contributions from Africa, **(E)** shows papers with author affiliations in all continents, and **(F)** shows papers with author affiliations from Africa.

Of the 2619 articles with first author affiliation country determined, only 52 (0.02%) had affiliations from institutions in Africa. Of the 1955 articles with last author affiliation country determined, only 43 (0.02%) had affiliations from institutions in Africa. A total of 1071 articles had all author affiliation countries determined, and 901 of these had >2 authors. Of the 901 articles with all author affiliation countries determined (>2 authors), only 44 (0.05%) have an affiliation in Africa (**Figure 2**).

### Collaborations

**Figure 3:**
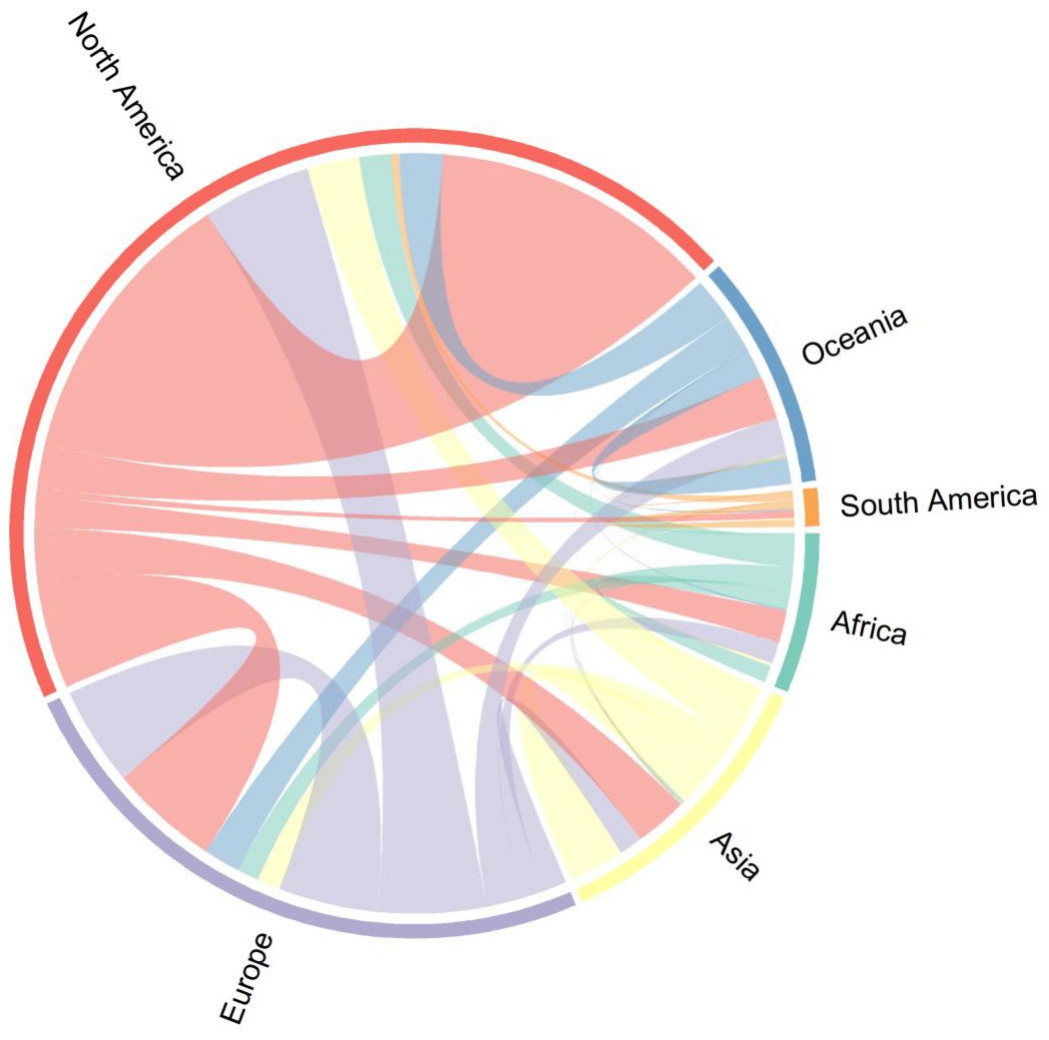
Intra and Inter-continental collaborations in HIV cure related research between 1995-2024.

Most of HIV cure related research has been conducted in North America through North-North collaborations, with only a handful of North-South collaborations as illustrated in **Figure 3**.

### Spectrum of HIV cure related research in Africa

**Figure 4:**
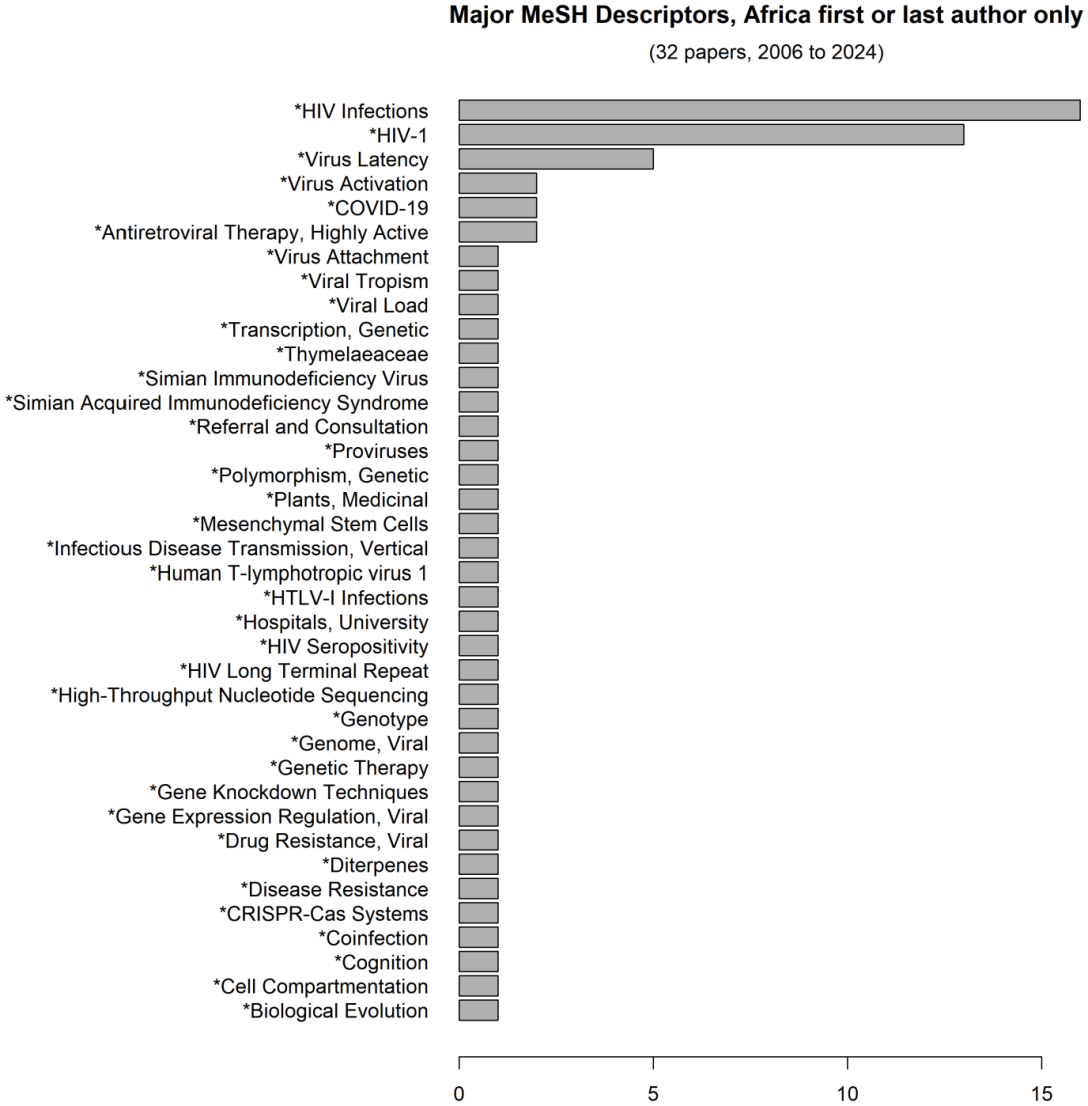
Scope of discovery HIV cure-related research in sub-Saharan Africa.

Most of the articles with Africa first or last authors were descriptive clinical studies of HIV infection, with less than ten studies specifically addressing HIV latency (**Figure 4**).

### Funding for HIV cure related research

**Figure 5:**
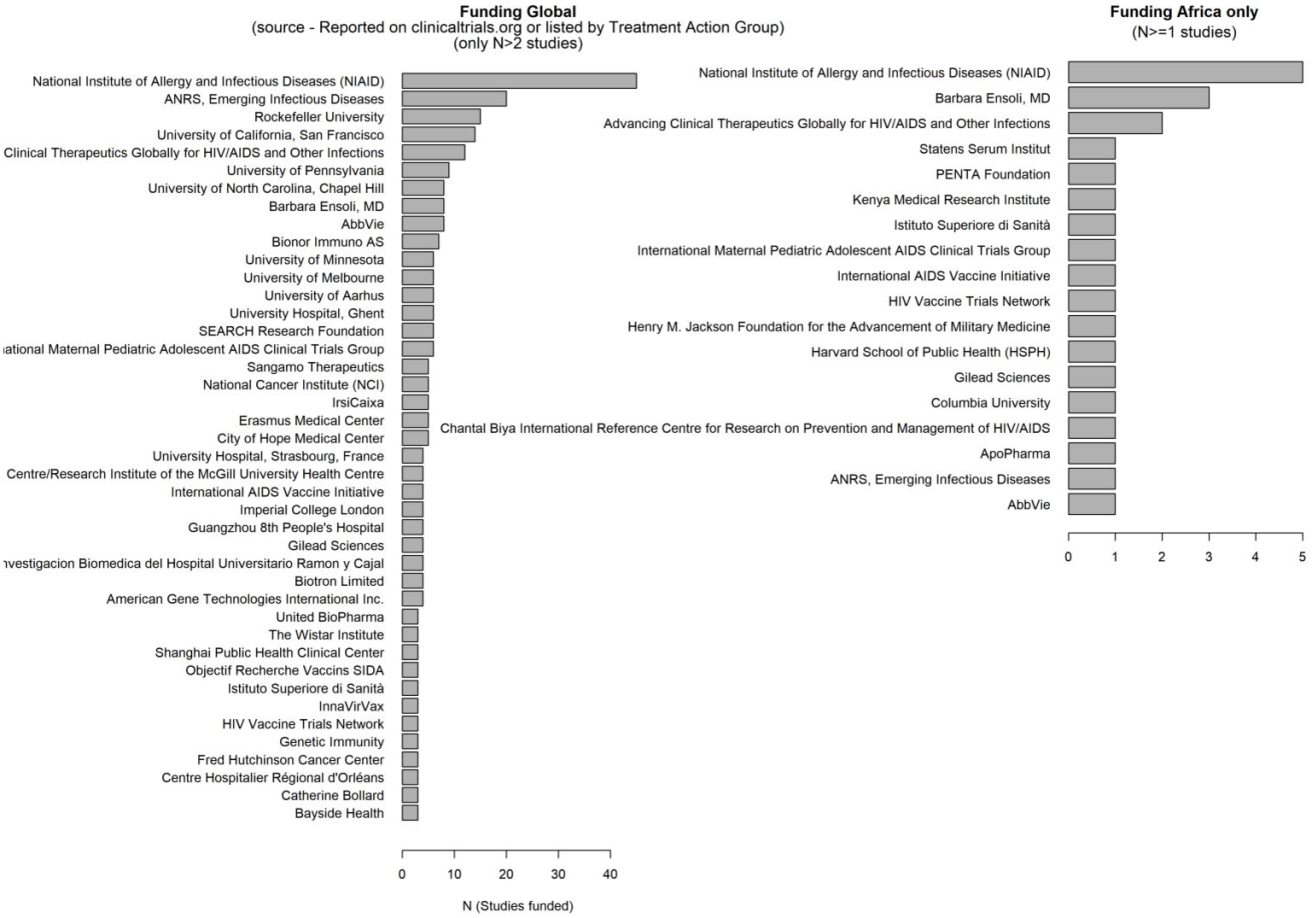
Sources of funding for HIV cure related research in sub-Saharan Africa between 1995-2024.

We observed limited funding for HIV cure related research in Africa between 1995-2024, and these few studies have been funded mostly by the National Institutes of Health in the United States of America (**Figure 5**; **Supplementary File**).

## Discussion

### Low numbers of HIV cure related research

The limited engagement in HIV cure research in Africa may slow the global progress towards an effective, durable and scalable HIV cure (28). Sub-Saharan Africa bears a disproportionately large burden of HIV, accounting for a majority of the global HIV-positive population despite having only a small percentage of the world’s population (1). The limited scope of HIV cure research in Africa is likely due to limited expertise, technology, research infrastructure and funding to conduct sustained HIV cure related biomedical research in the last three decades of the HIV epidemic in Africa. We report limited funding for HIV cure research in Africa. Moreover, most of the biomedical research reagents and products need to be imported from abroad which makes the cost of biomedical research considerably higher in Africa due to large incremental shipping costs and taxes before the products get to the laboratory bench. Many procurement systems in Africa have inefficient cold chain provisions for cell biology research and inefficient bureaucratic rather than technical supplier considerations, thereby adding extensive delays and compromising quality of temperature sensitive reagents (31). Similarly, the limited output of cure research in Africa is largely due to a limited critical mass of scientists with the capacity to conduct the required biological research; hence the need for intentional programs for capacity building, to train and contribute to the development of African scientists in the field (32).

### Narrow focus of HIV cure research

Regarding the spectrum of HIV cure related research in Africa in the last three decades, outputs were largely observational, descriptive clinical studies and drug treatment trials, with limited pre-clinical biomedical studies to understand the non-B HIV-1 subtypes that are prevalent in Africa. A few studies in southern Africa have been conducted on *in-vitro* reactivation of latent HIV-1 by cytostatic bis(thiosemicarbazonate) gold(III) complexes including two cytostatic bis(thiosemicarbazonate) gold(III) complexes (designated 1 and 2) that were tested for reactivation potential in the U1 latency model of HIV-1 infection (33). Other examples included a collaborative study between the University of Zurich, Switzerland and University of Pretoria, South Africa that was conducted in humanized mice to determine if gene-engineered CD34(+) hematopoietic stem and progenitor cells (HSPCs) can be used to generate an HIV-1-resistant immune system (34); and a collaborative study between the Scripps Research Institute, La Jolla, California, USA, Beckman Research Institute at the City of Hope, Duarte, California, USA and the University of the Witwatersrand, Johannesburg, South Africa to understand potent and targeted activation of latent HIV-1 using the CRISPR/dCas9 activator complex (35, 36). Gaps in knowledge, research and discovery remain in Africa largely due to limited facilities for pre-clinical studies that would promote a more comprehensive understanding of the non-B HIV subtypes. For example, strengthening pre-clinical research which provides crucial evidence that underpins scientific advances of HIV cure interventions that affect the reservoir, viral reactivation during ART, or viral recrudescence after ART interruption (28). Whereas immunotherapies including therapeutic vaccines, broadly neutralizing antibodies, immunomodulators and latency reversal agents are showing promising results in post-ART viral control, these trials need to be expanded in Africa to target two thirds of the world’s HIV epidemic. Similarly, promising technologies for cell and gene therapies including gene editing with CRISPR/Cas9 (or related) technology and cell-based therapies using chimeric antigen receptor (CAR)-T cells need to be promoted and increasingly tested in Africa, in the quest to achieve a sustained ART-free HIV remission (functional cure) or complete HIV elimination from the body, including from hidden reservoirs (sterilizing cure) (27, 28).

Furthermore, a major impediment to purging the reservoir is the limited understanding of the phenotypic characteristics of the reservoir virus. In subtype B infections, predominant in the United States and Western Europe, reactivating viruses following ART interruption are highly type I interferon (IFN-I)-resistant (37). However, these critical data are lacking for non-B subtypes which may well have different profiles (38–42). In addition, there is need to understand HIV persistence in the presence of co-infections such as tuberculosis, hepatitis B virus (HBV) and Hepatitis C virus (HCV), malaria and helminths that are endemic in sub-Saharan Africa. For example, development of single-cell level quantitation of distinct viral products serving as biomarkers for viral persistence such as inducible msRNA for HIV and cccDNA for HBV would be useful to conduct detailed characterizations within cohorts of HIV/HBV co-infection (43). There is therefore a need for strategic funding to bridge the infrastructure and technology divide between the global north and global south to empower scientists in the global south to conduct biomedical studies characterizing HIV disease pathogenesis and contribute to research and discovery towards an HIV cure for all (44). In addition, scientists in low-and-middle income countries (LMICs) should be supported to take on active roles in collaborative research that promote training, technology expertise and transfer, as well as relevant product discovery in Africa; to hasten achievement of the global goal of an HIV cure for all in the near future (45). This calls for strategic investment in sustainability of equitable, ethical and mutually beneficial interdisciplinary and intersectoral local, regional and global collaborations (46, 47).

### Limited funding for HIV cure research

Our results demonstrate limited funding for HIV cure research with most of the funding linked to the United States National Institute of Health divisions. In a bid to improve the contribution of the global south towards an HIV cure, the Johns Hopkins Centre for AIDS Research (CFAR) Africure program encourages early-career scientists in LMICs to apply for funding and lead relevant HIV cure studies in Africa (48). Consistent core funding of HIV cure-focused cohorts remains a challenge, with only a few examples that have been sustained for several years. The National Institute of Allergy and Infectious Diseases (NIAID)/Rakai Health Sciences Program (RHSP) Uganda Latent Viral Reservoir study features longitudinally collected peripheral blood mononuclear cells and plasma samples from 90 participants on suppressive ART that have been collected over 10 years as at 2025, and analyzed for several viral and host characteristics relevant to informing a cure strategies (11, 49). Between 2020 to 2025, the Research Enterprise to Advance a Cure for HIV (REACH), a Martin Delany Collaboratory, collaborated with Ugandan scientists to support training and technology transfer of innovative assays like the modified Intact Proviral DNA Assay (IPDA) for non-B HIV subtypes (50). Such opportunities need to be increased. Noteworthy is the fact that efforts to increase local and international funding for HIV cure related research should complement all ongoing initiatives to increase funding from African governments to support the most pressing health challenges that include endemic infections such as tuberculosis, HIV, malara as well as preparedness for pandemics. It is therefore essential for African countries to further increase the allocation of budgets and human resources to implement HIV-1 treatment and prevention interventions and equally invest in training and development of virologists in African countries, and increase capacity to carefully design and implement evidence-based strategies for significantly reduce the number of HIV-1 infections in Africa in the future. The Global Fund is proof that by working hand-in-hand with civil society, governments, private sector partners, philanthropists, technical partners and communities affected by the diseases, we can save lives and dramatically change the course of HIV, TB and malaria (51). Such initiatives need to be increased.

### Capacity building for HIV cure research in Africa

Despite the global commitment towards a world free of HIV (45), inequities continue to hinder global progress given the limited contribution from sub-Saharan Africa which hosts more than two-thirds of global population of PLHIV. There is need to support more Africa-based capacity building programs such as the Makerere University-Uganda Virus Research Institute Infection and Immunity research program - https://www.muii.org.ug/ (32) and the Sub-Saharan Africa Network for TB/HIV Research (SANTHE) - https://www.santheafrica.org/ (52), that aim to train and increase the critical mass of translational scientists in the fields of HIV cure and other endemic infections. SANTHE’S initiatives include the SANTHE HIV Cure Acceleration Research Programme (SHARP), which aims to address key research and capacity gaps in HIV cure research within the African continent. Similarly, strategic networks like African Research Universities Alliance (ARUA) need to strengthen their efforts to build regional biomedical research and discovery in Africa through the hub and spoke model (53). Synergy for cure research needs to be built within ARUA and other collaborative bodies outside Africa like the Guild of research universities in Europe that aim to promote graduate training and research at doctoral level, student-and-faculty mobility and technology transfer to bridge the inequities and increase the critical mass of skilled scientists to respond to emerging and re-emerging challenges to global health (54). Furthermore, regional collaborative initiatives through Africa Center for Diseases Control need to advocate for African governments to support HIV cure research initiatives so that Africa is not left behind in the current quest for AIDS eradication by 20230 and the search for a cure (44). These recommendations are in-line with the 2021 International AIDS Society global scientific strategy which highlights that beyond the science, key pre-requisites to attaining an effective, affordable and scalable HIV cure for all include efforts to strengthen international collaborations to ensure a multidisciplinary approach to HIV cure research in diverse settings, building capacity for HIV cure research in different populations, and increase involvement of researchers from countries most affected by HIV (28).

### Limitations

Some articles, especially the older articles, lacked proper author affiliation information. To determine author information, we considered the first listed affiliation as the main affiliation, although some authors may have multiple affiliations across different continents. We therefore recommend training in adequate documentation of author affiliation to improve monitoring and evaluation of progress and impact of HIV cure research, and other key global health challenges in Africa. It is also likely that contributions of African scientists were underestimated, particularly in consortia where several contributors may not get to the author list. There is need for better structured ways to identify, facilitate and recognize these contributions in-line with ethical guidelines for health system research and practice when working at a social or physical distance (55, 56). Furthermore, we acknowledge the bias associated with PubMed indexing and we minimized this by using the hybrid environmental scan which combined multiple information sources relevant to HIV cure beyond published academic literature.

## Conclusion and recommendations

Scale up of HIV cure research in sub-Saharan Africa, which hosts over two-thirds of the global HIV epidemic, is critical to hasten the achievement of the global goal of an end to the AIDS epidemic by 2030 that was set by the World Health Organization member-countries in 2016. There is a need to bridge technical, infrastructural, technological, capacity and funding gaps in Africa to promote pre-clinical studies on innovative therapies including immune therapies, cell and gene therapies directed towards the extensive viral subtype diversity and genetic heterogeneity in Africa. Beyond the science, we recommend strategic interventions to advocate for African scientific leadership, African-led discovery science, data sovereignty, as well as ethical and mutually beneficial collaboration to hasten the progress towards the global goal to attain an effective, durable, affordable and scalable HIV cure.

## Supplementary material

Supplementary Data Sheet 1 Detailed scope and funding for HIV cure related research in Africa between 1995-2024.
