# Supplementary figures and images for: HIV cure research contributions from Africa in the last three decades
Source: Front Immunol. 2025 Aug 8;16:1576667. doi: 10.3389/fimmu.2025.1576667 (PMC12370771; doi:10.3389/fimmu.2025.1576667)

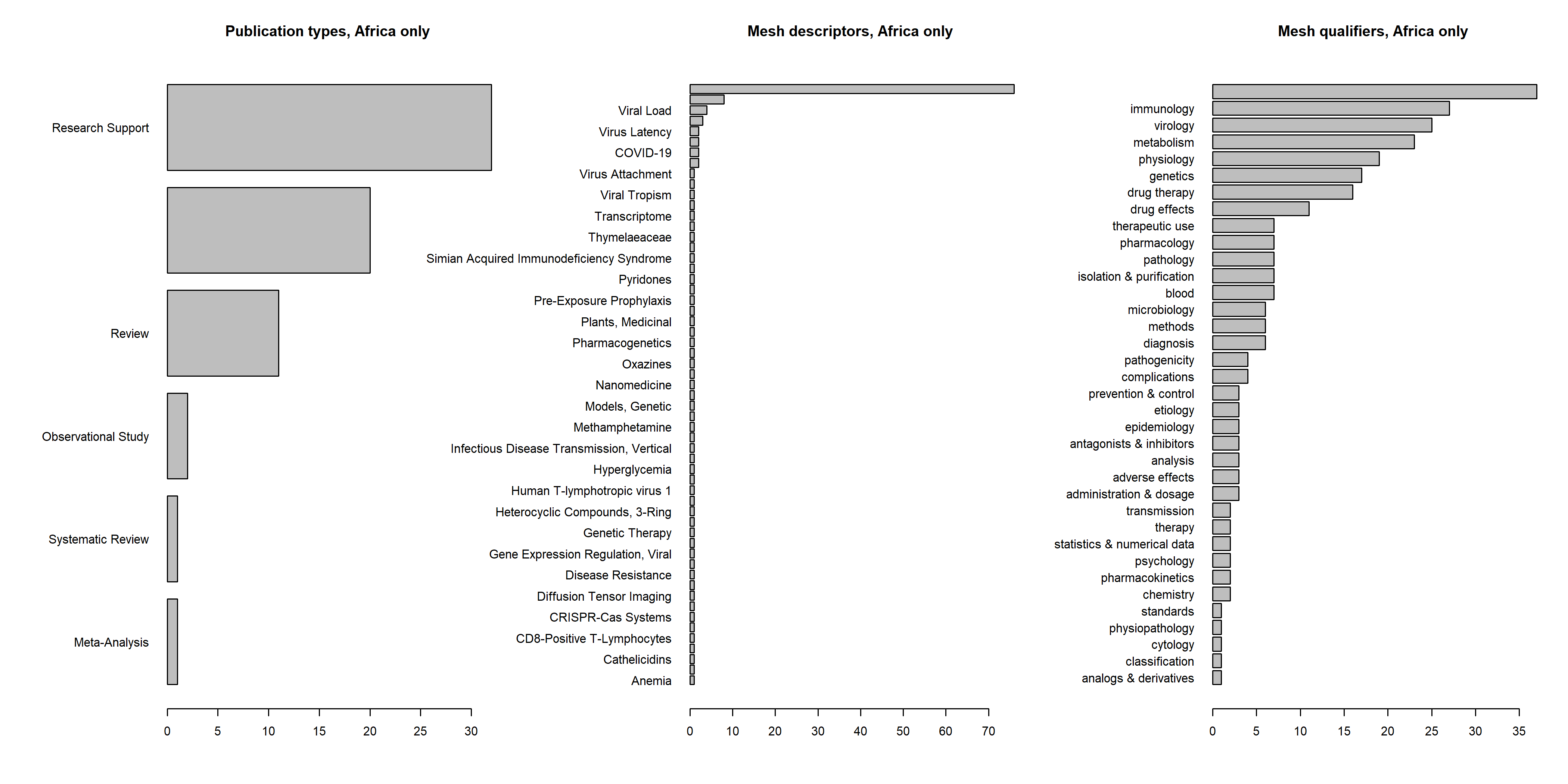


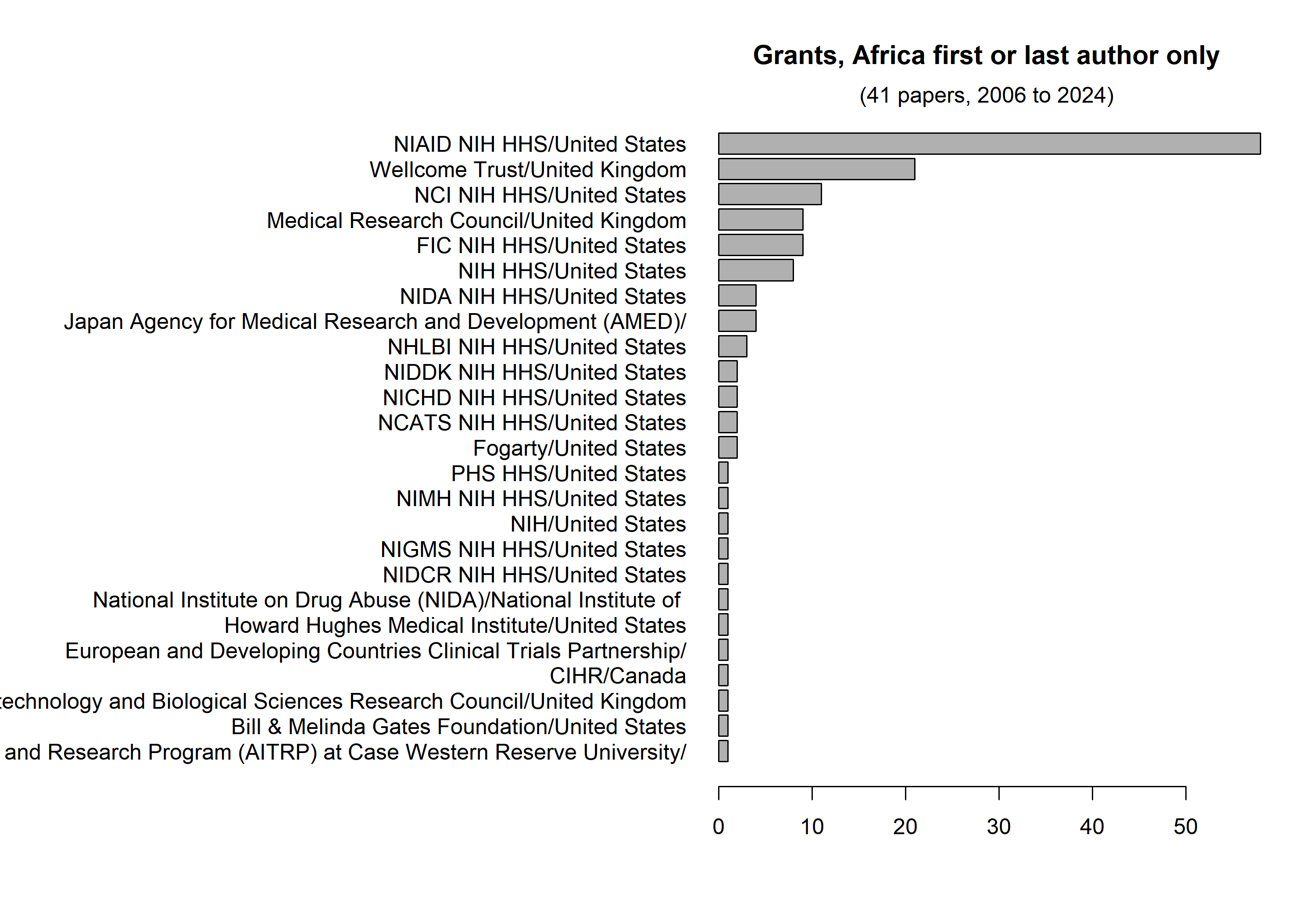

Supplement: Supplementary Data Sheet 1 — Detailed scope and funding for HIV cure related research in Africa between 1995-2024. [file DataSheet1.docx]
